# A simple and efficient quasi 3-dimensional viscoelastic model and software for simulation of tapping-mode atomic force microscopy

**DOI:** 10.3762/bjnano.6.229

**Published:** 2015-11-26

**Authors:** Santiago D Solares

**Affiliations:** 1 Department of Mechanical and Aerospace Engineering, George Washington University, Washington, DC 20052, USA

**Keywords:** atomic force microscopy (AFM), modeling, multifrequency, multimodal, polymers, simulation, spectroscopy, standard linear solid, tapping-mode AFM, viscoelasticity

## Abstract

This paper introduces a quasi-3-dimensional (Q3D) viscoelastic model and software tool for use in atomic force microscopy (AFM) simulations. The model is based on a 2-dimensional array of standard linear solid (SLS) model elements. The well-known 1-dimensional SLS model is a textbook example in viscoelastic theory but is relatively new in AFM simulation. It is the simplest model that offers a qualitatively correct description of the most fundamental viscoelastic behaviors, namely stress relaxation and creep. However, this simple model does not reflect the correct curvature in the repulsive portion of the force curve, so its application in the quantitative interpretation of AFM experiments is relatively limited. In the proposed Q3D model the use of an array of SLS elements leads to force curves that have the typical upward curvature in the repulsive region, while still offering a very low computational cost. Furthermore, the use of a multidimensional model allows for the study of AFM tips having non-ideal geometries, which can be extremely useful in practice. Examples of typical force curves are provided for single- and multifrequency tapping-mode imaging, for both of which the force curves exhibit the expected features. Finally, a software tool to simulate amplitude and phase spectroscopy curves is provided, which can be easily modified to implement other controls schemes in order to aid in the interpretation of AFM experiments.

## Introduction

The quantification of tip–sample dissipation in atomic force microscopy (AFM) has been an ongoing subject of interest since the early days of the technique [1–2]. A significant percentage of the surfaces characterized with AFM exhibit rate-dependent deformation processes that result in dissipative tip–sample interactions. A few examples of these processes include viscoelastic deformation, irreversible molecular structure changes (e.g., in biomolecules) and plastic deformation in crystals. These phenomena bring challenges into AFM characterization primarily in two ways. First, in delicate samples, such as biomolecules, it becomes necessary to control the maximum tip–sample interaction forces and stresses, such that undesirable irreversible changes do not occur in the sample. Second, the interpretation of the experiment requires the user to make assumptions and/or develop models that properly account for the rate-dependent dissipative processes.

Viscoelasticity, in particular, is a very difficult phenomenon to deal with accurately within AFM spectroscopy, whereby one tries to extract material properties following a set of measurements in which generally one parameter is varied while keeping all other parameters constant. The most common example of a spectroscopic measurement in AFM is the recording of an observable (e.g., phase shift, frequency shift, deflection, specific harmonic amplitudes, etc.), while the base of the microcantilever is brought closer to the sample with a relatively small constant speed, and then retracted at the same speed. Generally the desired information is the tip–sample interaction force curve, which for an elastic body is an analytical expression describing the force sensed by the AFM tip as a function of its vertical position above the sample. From this curve the user can extract properties such as the Young’s modulus, which describes the bulk stress–strain relation of the material, or the Hamaker constant, which describes the dispersion forces between the tip and the sample.

In the case of a viscoelastic surface the extraction of material ‘properties’ is difficult for a number of reasons. First, viscoelasticity itself is a difficult-to-quantify behavior at the nanoscale. In continuum measurements it is common to describe viscoelastic behavior in terms of the loss and storage moduli, but strictly speaking, these quantities are only meaningful in the case when a continuous periodic strain is applied to the sample and the probe–sample system is in steady state, which in AFM requires a contact-mode measurement such as contact-resonance AFM (CR-AFM) [3–5] or dual amplitude resonance tracking (DART) [4]. When the applied strain is not continuous and periodic, and the measurement process is not in steady state, it becomes extremely difficult to quantify viscoelastic behaviors in a meaningful way. Nevertheless, other authors [6–8] have very successfully implemented experimental intermittent-contact multi-frequency AFM methods that allow the extraction of analytical tip–sample interaction expressions in which the force is expressed as the sum of a Hertzian conservative interaction plus an indentation- and velocity-dependent dissipative interaction. Such 1-dimensional (1D) models have, for example, been used in the characterization of polymers [8–9], providing a modulus of elasticity and ‘dissipation’ parameters, which can be practical and efficient in a variety of situations. Nevertheless, further developments still remain in terms of model improvements that consider the most fundamental behaviors of viscoelastic bodies. Specifically, the above analytical models cannot reproduce stress relaxation and creep [10–11]. Within AFM, this means that when the tip and sample are held in contact at a fixed relative position*,* the model must exhibit a time-dependent reduction in the stress (stress relaxation). Additionally, when the tip and sample are held in contact at a fixed stress*,* the model must exhibit a time-dependent relaxation of the position of the sample directly under the tip. That is, the sample must yield, allowing the tip to gradually increase the depth of indentation. Furthermore, if the tip is quickly removed following yielding of the surface, the surface must remain depressed, with a cavity in it, and gradually relax afterwards. In particular, if the tip–sample interaction is of an intermittent contact nature, it may possible that the surface does not fully return to the original (undisturbed) position before the tip impacts it again. That is, during the second impact the tip may find the surface at a lower position than prior to the previous impact. These behaviors are discussed in detail in [10–11].

**Figure 1:**
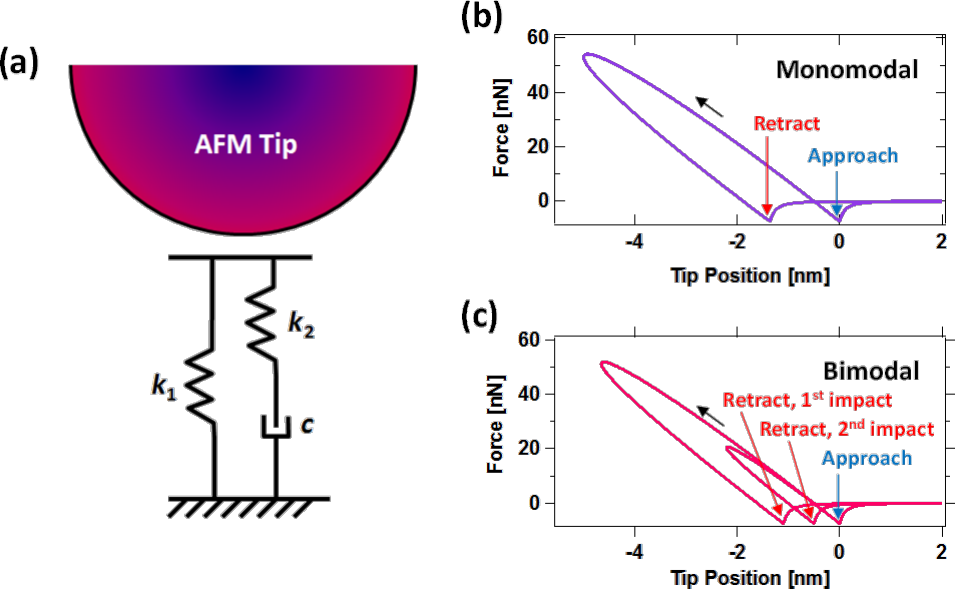
(a) Schematic of AFM tip interacting with the standard linear solid model; (b) example of force curve for monomodal AFM; (c) example of force curve for bimodal AFM, showing a double impact. The blue arrows indicate in each case the position where the tip first reaches the sample, and the red arrows indicate the position where the tip leaves the sample. Van der Waals forces have been included in the attractive (noncontact) region.

In an effort to provide a more fundamentally correct viscoelastic description of the surface, in recent intermittent-contact AFM studies we have used the 1D standard linear solid (SLS) model, which is a well-known textbook problem in viscoelasticity. The model is illustrated in Figure 1a and consists of a linear spring (*k*_1_) in parallel with a ‘Maxwell arm,’ which in turn consists of a linear spring (*k*_2_) in series with a linear damper (*c*). When a stress (force) or a strain (displacement) is applied to the model, spring *k*_1_ yields and generates a repulsive force that is proportional to the instantaneous displacement of the ‘surface.’ In the Maxwell arm spring *k*_2_ yields also producing a repulsive force, but in this case the force is proportional to the instantaneous displacement of the ‘surface’ minus the instantaneous displacement of the damper, which relaxes with a speed that is proportional to the instantaneous force generated by spring *k*_2_. The presence of the Maxwell arm, where complete relaxation of the stress (force) is possible, in parallel with the linear spring *k*_1_ allows the model to exhibit the desired viscoelastic behaviors, namely stress relaxation, creep, and also the ability to fully but gradually (not instantaneously) recover when all forces are removed. Additional details on stress relaxation and creep simulations are provided in [10]. Figure 1b and Figure 1c give examples of tip–sample force curves for intermittent-contact AFM in single- and multifrequency operation, respectively, when using the SLS model to represent the surface. As can be seen, the force curve shows separate force minima for the position where the tip first reaches the sample, and the position where it leaves the sample. These locations can be different due to creep of the surface. Furthermore, the model can be enhanced with multiple relaxation times by adding additional Maxwell arms (additional elements, each consisting of a linear spring in series with a damper), whereby these combined elements are placed in parallel with the SLS (a more complete description of these models and their advantages and disadvantages in the context of AFM is provided in [10–11]). Although the use of the SLS model in AFM is a step forward in terms of the physics of viscoelasticity, this linear model gives force curves that do not have the correct curvature in the repulsive region. It is clear in Figure 1b and Figure 1c that the force curve is concave downward instead of concave upward. The linear springs in the model lead to straight (linear) force curves, which become concave downwards as the surface creeps, via relaxation of the damper and spring *k*_2_. The incorrect curvature of the force curve is a serious shortcoming of the 1D SLS model within AFM, because it precludes the quantitative interpretation of the results of an experiment in terms of a real 3D tip interacting with a flat surface, and thus makes it impossible to extract approximate parameters such as the Young’s modulus [12]. It is clear in Figure 1a that the geometry of the tip and its indentation depth into the surface have absolutely no effect on the nature of the tip–sample interaction when a 1D model is used, unless the user explicitly programs geometric effects into the model, for example through the use of nonlinear springs [11].

In CR-AFM and DART [3–5] surface viscoelasticity is generally interpreted in terms of the Kelvin–Voigt model, consisting of a linear spring in parallel with a damper. This is appropriate (i) when the tip oscillation amplitude is very small, since in this regime the small segment of the force curve that is involved can be treated as quasi-linear, and (ii) when the tip and sample are in permanent contact (that is, the tip does not oscillate faster than the surface can relax). From this type of measurement one can extract storage and loss moduli, given proper calibration. The method has been enhanced by performing tomographic (volume) scanning [13], such that one can obtain the entire force curve via a 3D measurement. One can then analyze the depth dependence of the contact stiffness by performing a fit to appropriate models of elastic, viscous and adhesive forces, as is demonstrated in [13] for polymer blends. This approach is associated with small tip oscillations and is sensitive to the speed at which the base of the cantilever is approached towards and retracted from the sample. The method can be easily enhanced by relaxing the small oscillation amplitude requirement and using a variety of cantilever speeds to carry out the volume scan, although this may, in general, require the use of more complex tip–sample conservative–dissipative models within a simulation framework, in order to properly interpret the results.

If the highest accuracy is desired in AFM modeling, it is necessary to advance towards a model in which the various types of tip–sample interactions can be incorporated and tuned independently: long-range attractive forces (such as dispersion, electrostatic, magnetic), adhesive forces (such as chemical, capillary), viscoelastic forces, plastic forces, etc. For the case of viscoelasticity, in the most elaborate case one would need to solve the relaxation of the surface in 3D with the appropriate constitutive relation, as in the finite elements method (FEM), coupled with the dynamics of the cantilever. Given the number of research directions in which the AFM community is rapidly advancing, this may be unrealistic in terms of the knowledge and time required on the part of the user and in terms of computational cost. Nevertheless, it is important to gradually advance in that direction. To this end, the present paper introduces a quasi-3D (Q3D) surface model, along with a basic software tool, which consists of a 2D-periodic array of 1D-SLS models. This intermediate approach naturally incorporates important effects such as tip geometry effects (allowing for ideal and non-ideal tip shapes) and changes in the attractive forces due to changes in the surface geometry, following indentation and incomplete relaxation. Additionally, the Q3D model naturally leads to repulsive force curves that are concave upwards for spherical tips.

The subsequent sections of this paper provide (i) an overview the model features in the context of single- [12] and multifrequency [14–15] AFM characterization, (ii) a description of the simulation methodology, and (iii) a brief description of the software tool, which is provided as supplementary information.

## Results and Discussion

### Description and illustration of the Q3D model

**Figure 2:**
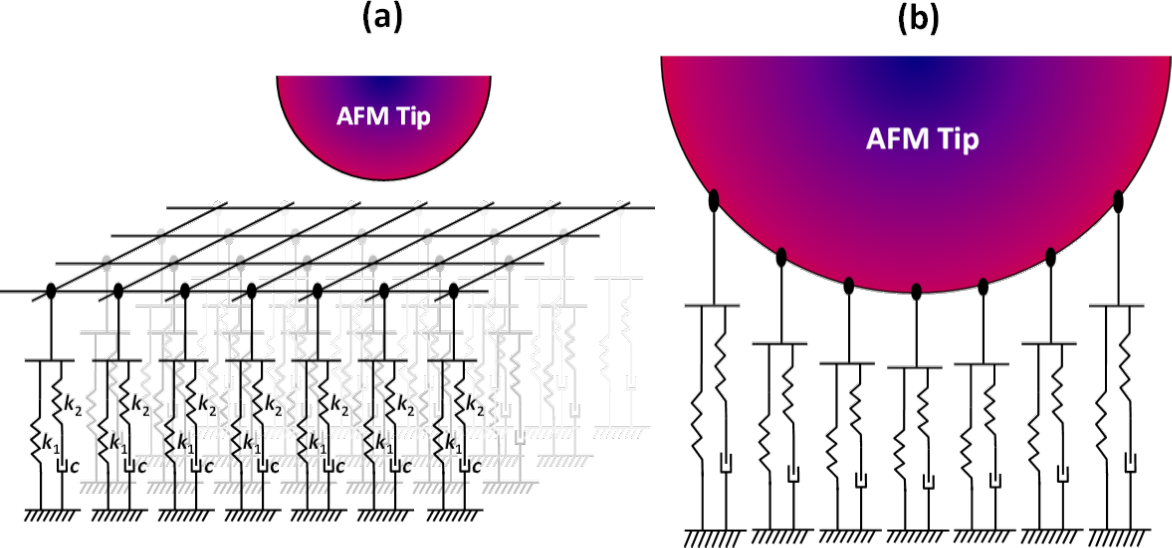
(a) Illustration of AFM tip approaching a 2-dimensional array of SLS models; (b) illustration of AFM tip interacting only with the SLS models directly below it, and interacting to a different depth with each element, as dictated by its geometry. Each SLS model in (a) and (b) is of the same form as the one shown in Figure 1a.

**Figure 3:**
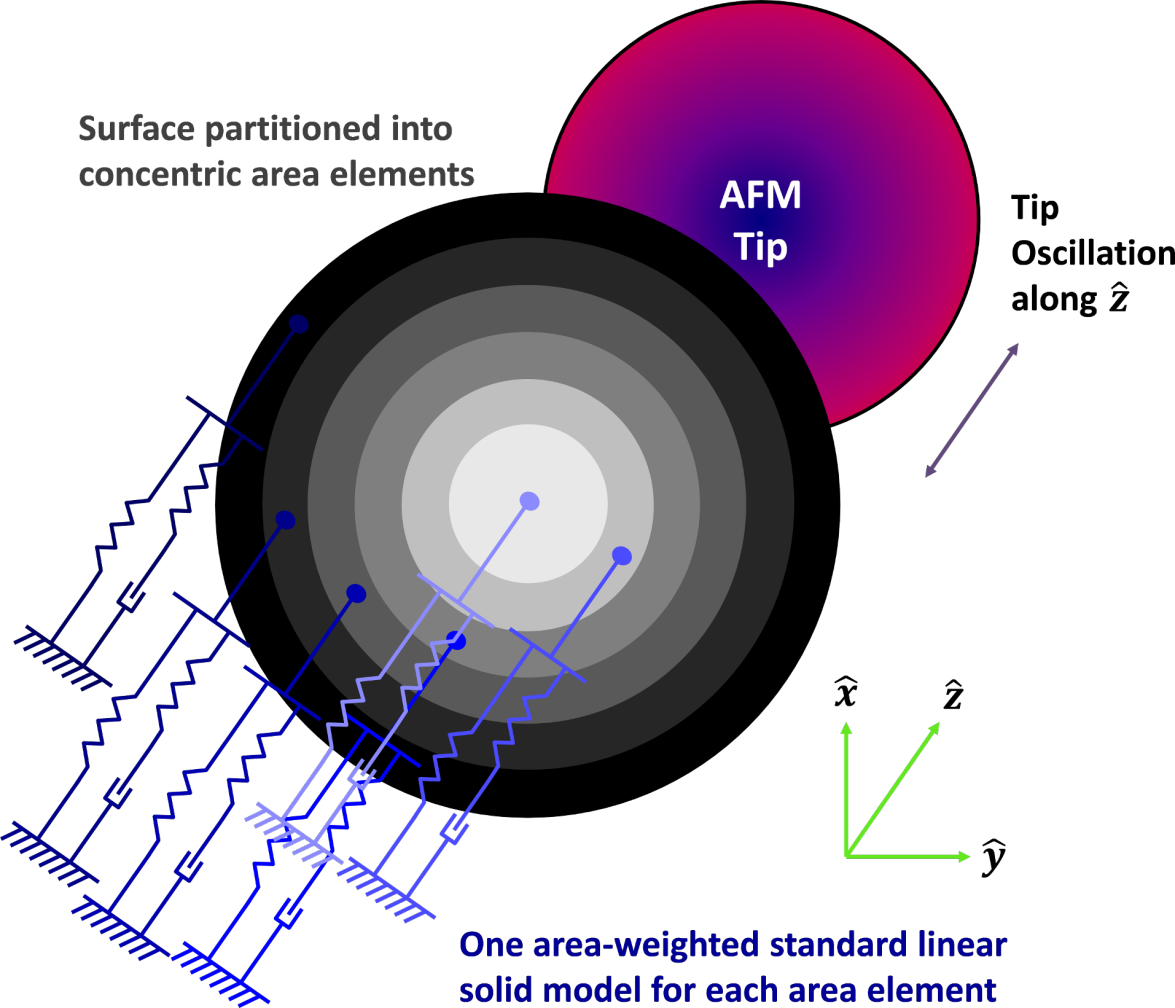
Illustration of the proposed model for a spherically symmetric AFM tip oscillating along the *z*-axis. Each concentric ring element is connected to an individual SLS model, whose parameters are proportional to the area of the ring.

The Q3D model consists of a 2D array of SLS models, as illustrated in Figure 2a. This is not a true 3D model since it is not based on a constitutive equation that describes the properties of the volume of material under the surface. Instead, it consists of ‘small’ SLS models distributed evenly in the *x*- and *y*-directions of the surface, each of which can relax independently in the *z*-direction upon interaction with the tip, which is modeled here as a hard sphere attached to the AFM cantilever. As depicted in Figure 2b, the degree of relaxation of each individual SLS model is dictated by the geometry of the tip. Given the spherical symmetry of the ideal AFM tip, it is convenient to use polar coordinates, whereby the surface is modeled as a set of concentric rings (Figure 3), in which the radial coordinate is partitioned into equal segments of length Δ*r*, and the width of each element is defined by Δ*r*. Additionally, we consider that each element is connected to a SLS whose parameters are proportional to its surface area, 
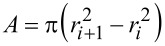
. This reduces significantly the computation time required to calculate the interactions of the model with the tip. However, the imposition of radial or any type of symmetry is not a requirement and any arbitrary distribution of SLS parameters over the 2D surface can be defined either in rectangular or polar coordinates. For brevity and simplicity this paper illustrates only the case of radially symmetric AFM tips and surfaces, including a defective tip that has a cluster protruding from its apex (this is described below). Similarly, the software tool provided assumes radial symmetry, but it can be easily modified to allow deviations from it.

**Figure 4:**
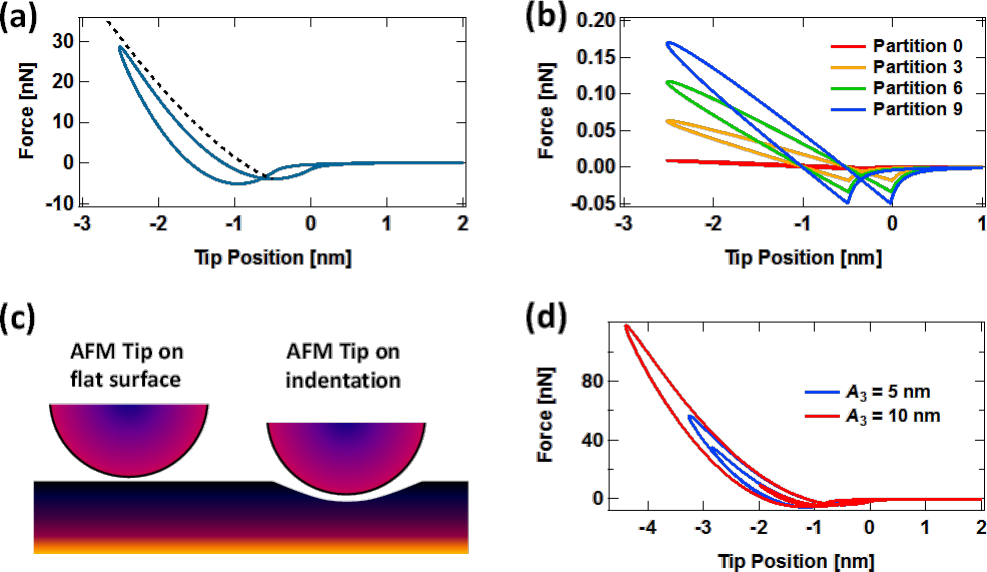
(a) Typical force curve for a spherical tip interacting with the Q3D surface model in monomodal tapping-mode imaging (the dashed line is a plot of a Hertzian curve, for reference); (b) illustration of the contributions to the force curve from different concentric-ring surface elements (numbered starting with the element that intersects the tip vertical axis): as the tip indents deeper into the sample, new surface elements of increasingly larger area become active and contribute to the force curve (recall that the SLS contribution of each surface element is proportional to its area); (c) schematic of the greater van der Waals interaction for a tip interacting with a cavity on the surface with respect to a tip interacting with a flat surface; (d) typical Q3D force curves for bimodal AFM imaging using the first and third eigenmodes. Note that the level of indentation increases as A_3_ increases. Note also the resemblance to the force curve shown in Figure 1c. For (a), (b) and (d) the cantilever was placed at a height of 75 nm above the surface and the following parameters were used: first free oscillation amplitude *A*_1_ = 100 nm, third free oscillation amplitude *A*_3_ = 5 and 10 nm (as shown in (d)), fundamental frequency ν = 70 kHz, fundamental force constant *k* = 4 N/m, eigenmode quality factors *Q*_1_ = 150, *Q*_2_ = 450, *Q*_3_ = 750; tip radius of curvature *R* = 20 nm, and SLS parameters (see Figure 1) *k*_1_ = *k*_2_ = 7.5 × 10^−2^ N/m/nm^2^, and *c* = 1.0 × 10^−7^ N s/m/nm^2^ (monomodal AFM) and 2.5 × 10^−8^ N s/m/nm^2^ (bimodal AFM).

Figure 4a shows a typical force curve for the Q3D model, which in contrast to the results provided in Figure 1, does have the correct qualitative (upward) concavity in the repulsive region, which occurs because the tip interacts with an increasing number of SLS elements as the indentation increases and the contact area grows. This is illustrated in Figure 4b, which shows an example of the tip–sample force contributions of different area elements that add up to give the total force. Additionally, similar to the SLS, the Q3D force curve shows the qualitatively correct relaxation of the surface, with the surface remaining depressed upon rapid retract of the AFM tip following each impact, depending on the model parameters (see discussion of Figure 1b above). Finally, it is worth noting that the force minima for the approach and retract have a different force magnitude in Figure 4a. This is caused by a temporary cavity that remains on the surface upon tip retract, such that depending on the SLS parameters chosen, this cavity partially encloses the tip as it leaves the sample. This allows the sample surface to interact closely with a larger portion of the tip, compared to a flat sample surface (see Figure 4c), leading to greater van der Waals attractive forces during tip retract (this is also discussed in [10]). Figure 4d shows two force curves for the Q3D model in multifrequency AFM operation for different higher mode amplitudes, which exhibit the expected qualitative features (compare to the curve in Figure 1c). As expected, a larger amplitude in the higher eigenmode also leads to greater indentation during each impact [16].

**Figure 5:**
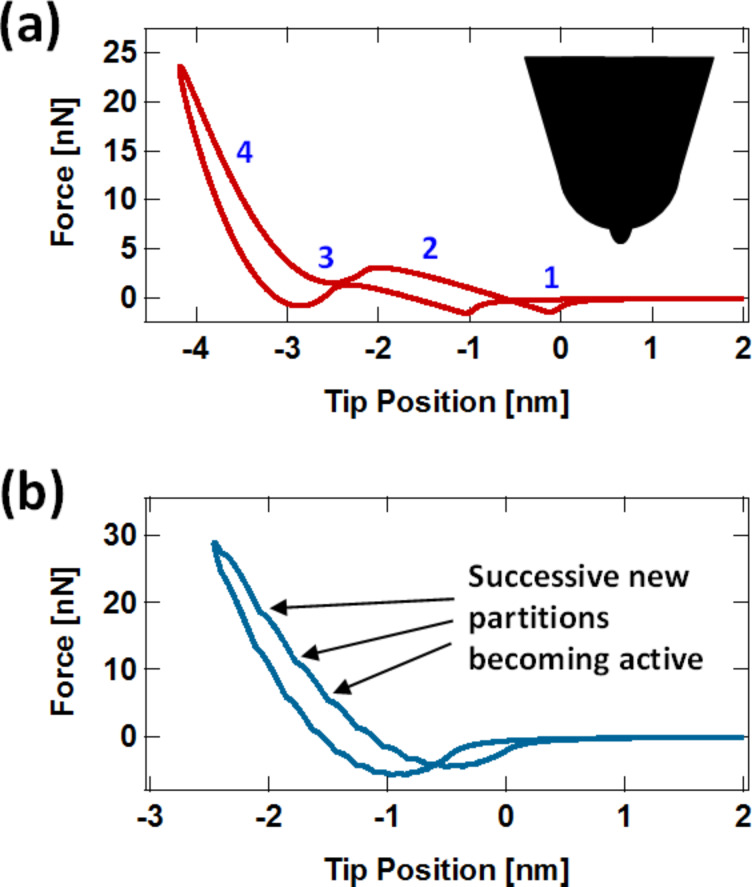
(a) Force curve for a 20 nm radius tip with a 2.5 nm radius protrusion at its apex as shown in the inset. The blue labels on the curve indicate the locations where, (1) the apex protrusion is approaching the surface in the attractive regime, (2) the apex protrusion is experiencing repulsive forces, (3) the rest of the tip is approaching the surface in the attractive regime, and (4) the entire tip is experiencing repulsive forces. The retract portion of the curve is similar to the approach but shows the expected offsets in the force minima, which are a consequence of viscoelastic relaxation. (b) Oscillations in the force curve due to the use of too coarse a surface partition. The simulation parameters are the same as for Figure 4, except for the irregular tip geometry in (a) and the coarser partition in (b) described in the text.

Even limiting the simulations to radially symmetric tips and samples, there is a wide range of phenomena that can be studied with the Q3D model, such as irregular tips, which are not uncommon. Figure 5a shows an force curve for a tip with a narrow protrusion at the apex, which leads to surprising anomalies, which at first glance may seem unreasonable. However, careful inspection leads to the eye-opening conclusion that this is not so: The region of the curve labeled with the number ‘1’ shows a small force minimum indicating that the apex protrusion is reaching the surface, experiencing van der Waals interactions, but has not yet reached the repulsive regime, which is labeled with the number ‘2’. Region ‘3’ indicates that the rest of the tip is approaching the surface and experiencing a significant attractive force that overcomes the repulsive regime from the small protrusion (this is reasonable because the tip is significantly larger than the protrusion). Finally, in region ‘4’ the entire tip and its apex protrusion are in the repulsive force regime. The retract portion of the curve is similar to the approach but has offsets in the two force minima due to relaxation of the surface, as previously discussed. There are other types of more subtle tip irregularities which are rarely considered in the literature, but which could be important in a quantitative study and which can be easily evaluated with the Q3D model (without losing sight of its limitations, as discussed below), such as slightly flattened tips or tips with a parabolic profile. Note, however, that anomalies in the calculated force curve may also be the result of non-optimized simulation parameters. For example, the force curve shown in Figure 5b exhibits a series of kinks that are caused by the use of a coarse surface partition (i.e., the concentric ring elements in the surface model are too large or, conversely, too few area elements have been used to describe the surface). The smooth force curves shown in Figure 4 were obtained with a partition where Δ*r* was set to (1/180)*R,* where *R* is the tip radius of curvature, while the curve of Figure 5b is based on a partition that is six times coarser.

An important consideration in the use of the Q3D model is the question of calibration against experimental observables. Since the force interactions that are obtained with the model can be highly dependent on the imaging parameters and the geometry of the tip, it is not generally possible to derive analytical expressions that provide the tip–sample force in terms of continuum properties. This is especially true for the intermittent-contact AFM case, where such analytical inversion is not possible even with the simple 1D SLS model, as discussed extensively in reference [10]. Nevertheless, to aid in the interpretation of experiments it is possible to carry out calibration procedures in which an experiment is performed and the Q3D model parameters are adjusted to match the experimental observations. An example of this could be the construction of a frequency response curve (amplitude vs frequency) under different values of the static deflection (with the deflection setpoint fixed for every simulation), which can be directly compared to CR-AFM measurements carried out under the same conditions. This could be especially valuable if, in addition to the CR-AFM observables, an image of the tip geometry is available, which would allow for the incorporation of geometry effects into the simulations. A second type of calibration may be the acquisition of static force distance curves in which the deflection is measured while the cantilever approaches and retracts from the surface at a fixed speed. To enhance the calibration, a collection of such curves could be constructed at different cantilever speeds. These considerations on model calibration suggest that a useful avenue of research may be the study of tip–sample force ‘signatures’ for different viscoelastic models, as proposed through simulations in [17], where the tip–sample interaction force curve is acquired using spectral inversion methods [18–19] and the force is plotted not only in terms of position but in terms of both position and velocity (that is, the force is expressed as 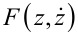 instead of simply *F*(*z*)). This enhanced representation may make it possible to invert the AFM observables to obtain viscoelastic model parameters. At this time this approach is still limited by experimental capabilities in the recording of the force spectrum [17,20] as well as by the lack of theoretical development required to infer viscoelastic model properties from such curves.

In order to place the Q3D model in the proper perspective it is important to discuss not only the advantages it offers, but also its shortcomings. The first shortcoming derives directly from its simplicity and computational efficiency: since the individual SLS elements do not interact with one another, the model does not consider material relaxations in the horizontal directions. As a result, it cannot be used as a ‘first-principles’ simulation tool, but instead only as a fitted tool that requires calibration either via experiments or more elaborate calculations (e.g., FEM simulations). A second limitation, which is related to the above, is that the model surface has no internal cohesiveness. As a result, the indentation profiles at static deflection will always follow the shape of the tip. That is, the largest cavity that the tip can induce is equal to the size of the tip. This is not the case in practice for most surfaces, where the size of the cavity is often expected to be larger than the diameter of the tip. To understand this, consider an AFM tip that is a perfect cube and impacts the sample with one of its faces oriented parallel to the sample surface. Within the Q3D model the indentation will be a perfectly square hole with vertical side walls, with the perimeter of the hole being exactly the same as the square perimeter of the tip. In a real experiment, the side walls would not be perfectly vertical but would instead be tapered, giving a cavity that is wider than the cross section of the tip. The Q3D model becomes less realistic for very large indentations, near and beyond the tip radius of curvature, and for very sharp tip geometries. These limitations can be partially mitigated by adding additional viscous and elastic elements between adjacent surface locations, although these would come with an added computational cost.

## Experimental

### Cantilever dynamics modeling

The dynamics of the AFM cantilever were modeled as in previous studies [16] using one equation of motion for each of the first three eigenmodes, whereby the three equations are simultaneously integrated numerically, coupled through the tip-sample force. Each equation is of the form

[1]
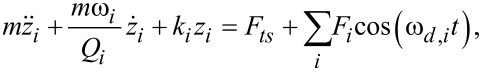


where *m* is the cantilever mass, *z_i_* is the eigenmode displacement as a function of time, ω*_i_* is the resonance frequency of the eigenmode, *Q_i_* its quality factor and *k_i_* its dynamic force constant. Additionally, *F*_ts_ is the total tip–sample force and the last term on the right hand side is the sum of the sinusoidal driving forces included for the various eigenmodes. Each term consists of an excitation force amplitude (*F_i_*) and a cosine term that depends on the respective excitation frequency ω*_d,i_* and time *t*. Excitation force terms were included for all three eigenmodes, each matching the corresponding eigenfrequency and having a magnitude that yields the desired free oscillation amplitude. The total tip–sample force term *F*_ts_ consists of the repulsive forces generated by the Q3D model (these are calculated numerically since there does not exist an analytical expression to calculate them [10]) plus attractive van der Waals forces, which are included for each area element in the Q3D model via an equation similar to the Hamaker equation [12]. Thus, the contribution to the van der Waals forces for area element *j* is

[2]
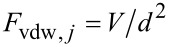


where *V* is a van der Waals ‘strength’ parameter in the code (see c-file in Supporting Information File 1) that adjusts the magnitude of the van der Waals interaction between each individual SLS element and the tip, and *d* is the distance between element *j* and the tip surface. The amplitude and phase of each eigenmode were calculated using the in-phase (*I_i_*) and quadrature (*K_i_*) integrals:

[3]
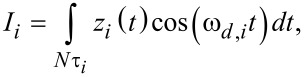


[4]
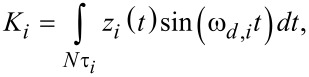


where *z_i_*(*t*) is the eigenmode response in the time domain, as in Equation 1, *N* is the number of periods over which the phase and amplitude were averaged, ω*_d,i_* is the excitation angular frequency, and τ*_i_* is the nominal period of one oscillation of the eigenmode. The amplitude *A_i_* and phase 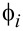 were calculated, respectively, as:

[5]
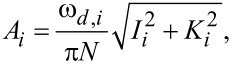


[6]
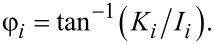


### Software tool description

**Figure 6:**
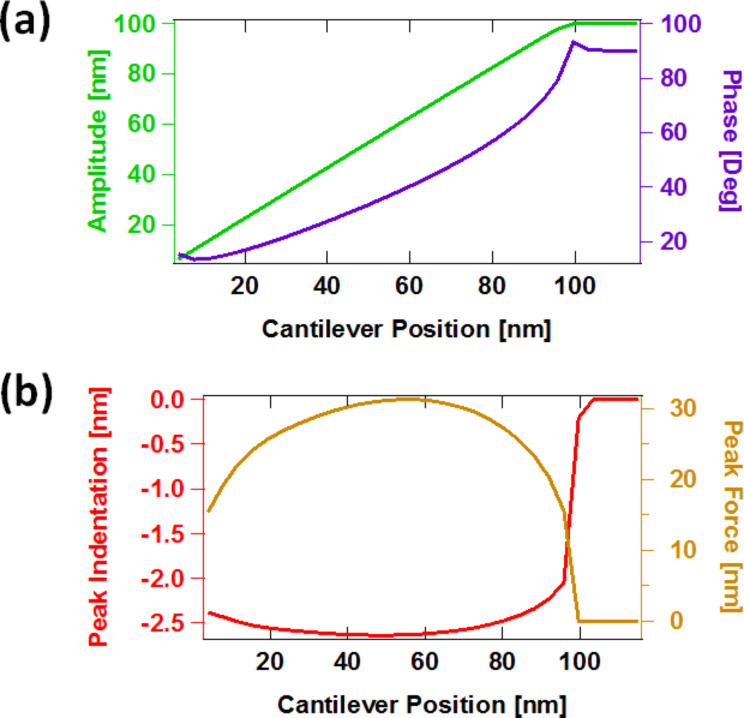
Examples of spectroscopy curves: (a) amplitude and phase vs cantilever position; (b) peak indentation and peak force vs cantilever position (these two quantities are not directly observable in a spectroscopy experiment). The simulation parameters are the same as for monomodal AFM in Figure 4.

The software tool, written in standard C programming language, provided within Supporting Information File 1, consists of an implementation of the above multifrequency (trimodal) cantilever dynamics in the construction of a point-by-point amplitude-modulation (AM-AFM) spectroscopy curve (amplitude and phase vs cantilever height), although other controls schemes as well as line scanning can easily be implemented, depending on the problem under study. To construct the spectroscopy curve, the user edits an input file which must be located in the same directory as the program executable file and contains the output file root name, the fundamental frequency and force constant (the higher-order frequencies and force constants are estimated based on an ideal rectangular cantilever), the first three quality factors, the starting height of the cantilever above the sample (at the beginning of the spectroscopy experiment), the target oscillation amplitudes for the three eigenmodes and the SLS parameters normalized by surface unit area. The software then performs a simulation in which the cantilever is set at successively lower heights above the surface and driven until it has reached steady state at each height. At this point, calculation of the phase and amplitude begins along with recording of the data in the output files. The program creates one output file for each cantilever height, which contains the most relevant dynamic information (such as time, instantaneous tip position, instantaneous value of each eigenmode coordinate, instantaneous tip–sample force, instantaneous amplitude, phase). In addition, the program also produces a second output file at the end of the run, which contains the amplitude, phase, peak force and peak indentation recorded for each value of the cantilever height. Figure 6 provides an example of the spectroscopy data obtained, which exhibits the expected features [12,16].

A variety of comments are provided throughout the code to aid the user in following the logic. Thus, it is quite easy to modify settings such as the settling time, printstep, the desired quantities in the output files, timestep (a reduction of the timestep should be considered for cantilevers with very high fundamental frequencies, in the MHz regime), number of cantilever height points in the spectroscopy curve, etc.

During benchmarking on an Intel^®^ Xeon^®^ Processor E5-1660 v3 (3.0 GHz) the code completed an equilibrated run at a fixed cantilever height in approximately 120 min. Thus, the total time required to construct the full spectroscopy curve was approximately equal to 120 min times the number of points in the curve.

## Conclusion

A quasi-3D viscoelastic model, consisting of a 2D array of standard linear solid elements has been proposed for the simulation of AFM imaging of viscoelatic surfaces. An efficient and easily modifiable software tool for the construction of amplitude and phase spectroscopy curves has also been provided as Supporting Information. The model correctly reproduces the key features of tip–sample interaction force curves acquired on a sample that exhibits stress relaxation and creep. In particular, the model qualitatively reproduces the upward curvature of the force curve in the repulsive region, as well as the relaxation and magnitude variation of the attractive force minima, which are a consequence of temporary variations in the surface geometry, following indentation by the tip. The model is a step forward in terms of introducing more accurate physics into the modeling of viscoelastic soft matter within AFM while keeping the computational cost relatively low, and can be further enhanced through the introduction of additional springs and dampers connecting adjacent SLS elements, through the use of 1D models with more than one relaxation time, or through the use of nonlinear elements [11].

## Supporting Information

Supporting Information consists of a ZIP archive containing three files: A program manual (Trimodal_AFM_with_Quasi3D_SLS+-+Files+Description.pdf) describing the content of the software files and their usage, the program source file written in C programming language (Trimodal_AFM_with_Quasi3D_SLS.c) and the input file for user-defined parameters (input.txt).

File 1 Program sources and manual.
