# Supplementary material for: A simple and efficient quasi 3-dimensional viscoelastic model and software for simulation of tapping-mode atomic force microscopy
Source: Beilstein J Nanotechnol. 2015 Nov 26;6:2233–41. doi: 10.3762/bjnano.6.229 (PMC4685832; doi:10.3762/bjnano.6.229)
Supplement: File 1 — Program sources and manual. [file Beilstein_J_Nanotechnol-06-2233-s001.zip › Trimodal_AFM_with_Quasi3D_SLS+-+Files+Description.pdf]

Supporting Information

for

A simple and efficient quasi 3-dimensional viscoelastic model and software for simulation of tapping-mode atomic force microscopy

Santiago D. Solares

Address: Department of Mechanical and Aerospace Engineering, George Washington University, Washington, DC 20052, USA

Email: Santiago Solares - ssolares@gwu.edu

Program manual

DESCRIPTION OF SOFTWARE FILES PROVIDED

The software tool provided consists of the following two files:

1. **'Trimodal_AFM_with_Quasi3D_SLS.c'** (source code written in C language, ready to be compiled)
2. **'input.txt'** (input file)

In order to execute the code, it is necessary to compile the first file to create the executable, and place the second file in the same directory. The user may edit the file 'input.txt' in order to provide the desired simulation parameters.

CONTENT OF THE INPUT FILE

The input file allows the user to specify the following parameters (the units are specified in parenthesis when applicable):

1. **OutputFile_1:** Root name for the output files.
2. **Frequency_1 (Hz):** Cantilever fundamental eigenfrequency.
3. **ForceConstant_1 (N/m):** Cantilever fundamental force constant.
4. **QualityFactor_1:** Cantilever fundamental quality factor.
5. **QualityFactor_2:** Second eigenmode quality factor.
6. **QualityFactor_3:** Third eigenmode quality factor.
7. **CantileverRestPosition (nm)**: Initial rest position of the cantilever during the spectroscopy curve.
8. **TargetAmplitude_1 (nm):** *Free* amplitude of the fundamental eigenmode.

9. **TargetAmplitude_2 (nm):** *Free* amplitude of the second eigenmode.
10. **TargetAmplitude_3 (nm):** *Free* amplitude of the third eigenmode.
11. **k_1 (N/m/nm^2):** Residual spring constant of the SLS model (see Figure 1 of the main paper).
12. **k_2 (N/m/nm^2):** Second spring constant of the SLS model (see Figure 1 of the main paper).
13. **cDiss (Ns/m/nm^2):** Dashpot constant of the SLS model (see Figure 1 of the main paper).

In addition, the user may want to modify the following variables in the code (the location is indicated in parentheses):

1. **TipRadius ('CONSTANTS' section at the beginning of the code):** Radius of curvature of the AFM tip.
2. **PartitionSize ('CONSTANTS' section at the beginning of the code):** Variable defining the coarseness of the surface partition (a larger value creates a finer partition and vice-versa).
3. **NoPoints ('CONSTANTS' section at the beginning of the code):** Number of points (different cantilever positions) desired in the spectroscopy curve.
4. **VdwStrength ('CONSTANTS' section at the beginning of the code):** Variable defining the strength of the van der Waals interaction between the tip and surface.
5. **timestep (main program):** Timestep for the integration of the cantilever dynamics and relaxation of the surface.
6. **startprint (main program):** Time at which printing of the output files begins, defined in terms of the fundamental quality factor and period.
7. **printstep (main program):** Time increment at which the data in the output file is printed, defined in terms of the fundamental period.
8. **length (main program):** Total length of each run at each fixed cantilever height, defined in terms of the fundamental quality factor and period.

OUTPUT FILES

As stated in the main paper, the software performs a spectroscopy curve simulation in which the cantilever is set at successively lower heights and driven while it interacts with the surface, until it has reached steady state (this condition is defined by the variable 'startprint'). At this point, calculation of the phase and amplitude begins, along with printing of the output. The program creates one output file for each equilibration of the cantilever at each fixed height (the name of this output file indicates the cantilever height to which it corresponds), which contains the most relevant dynamics information (time, instantaneous tip position, instantaneous value of each eigennmode coordinate, instantaneous tip-sample force, instantaneous amplitude, phase, etc.). In addition, the program also produces a second output file upon completing the spectroscopy curve (the name of this output file ends with 'ForceDepth'), which contains the amplitude, phase, peak force and peak indentation recorded for each value of the cantilever height.
